# Nano-printed miniature compound refractive lens for desktop hard x-ray microscopy

**DOI:** 10.1371/journal.pone.0203319

**Published:** 2018-08-30

**Authors:** Mona Mirzaeimoghri, Alejandro Morales Martinez, Alireza Panna, Eric E. Bennett, Bertrand M. Lucotte, Don L. DeVoe, Han Wen

**Affiliations:** 1 Imaging Physics Laboratory, Biochemistry and Biophysics Center, National Heart, Lung and Blood Institute, National Institute of Health, Bethesda, Maryland, United States of America; 2 Department of Mechanical Engineering, University of Maryland, College Park, Maryland, United States of America; 3 Cardiac Energetic Laboratory, National Heart, Lung and Blood Institute, National Institute of Health, Bethesda, Maryland, United States of America; Pennsylvania State Hershey College of Medicine, UNITED STATES

## Abstract

Hard x-ray lenses are useful elements in x-ray microscopy and in creating focused illumination for analytical applications such as x-ray fluorescence imaging. Recently, polymer compound refractive lenses for focused illumination in the soft x-ray regime (< 10 keV) have been created with nano-printing. However, there are no such lenses yet for hard x-rays, particularly of short focal lengths for benchtop microscopy. We report the first instance of a nano-printed lens for hard x-ray microscopy, and evaluate its imaging performance. The lens consists of a spherically focusing compound refractive lens designed for 22 keV photon energy, with a tightly packed structure to provide a short total length of 1.8 mm and a focal length of 21.5 mm. The resulting lens technology was found to enable benchtop microscopy at 74x magnification and 1.1 μm de-magnified image pixel size at the object plane. It was used to image and evaluate the focal spots of tungsten-anode micro-focus x-ray sources. The overall system resolution with broadband illumination from a tungsten-anode x-ray tube at 30 kV and 10 mm focal distance was measured to be 2.30±0.22 μm.

## Introduction

Microscopy in the hard x-ray photon energy regime (>10 keV) can take on a number of basic designs, such as projection magnification in a cone-beam, geometric magnification in a pinhole camera, and magnification based on a focusing element. The use of a focusing element offers several advantages by obviating the need for a small source spot for its resolution, allowing direct imaging from a light emitting source such as the focal spot of an x-ray tube, and supporting higher x-ray flux than a pinhole camera for faster imaging.

Focusing elements for x-ray microscopy up to 10 keV photon energy include Kirkpatrick-Baez mirrors in benchtop microscopy [1], zone plates at 8 keV [2] and compound refractive lenses (CRL) which consist of linear arrays of individual refractive lenses [3]. The first CRLs were lines of cylindrical holes drilled into aluminum blocks [3], where the small refractions of individual holes were compounded to achieve a substantial focusing effect [3]. Since this initial demonstration, CRL technology has been further developed through different designs, including parabolic [4], spherical [5], and kinoform [6] CRLs. Low-attenuation materials such as Al [3], Be [7], Si [8], C [9] and polymers[10][5] together with different fabrication techniques [11], have been explored.

The aim of this work it to develop a miniature CRL technology for benchtop x-ray microscopy in the 10s of keV energy range with high resolution and short exposure times. Since efficient detectors for hard x-rays have relatively large pixel sizes, high resolution implies the need for high magnification factors, which translates to short focal length of the focusing element. Furthermore, a larger numerical aperture is also desired to collect more light and shorten the exposure time. For a parabolic CRL of a fixed total length *L*, the focal length *f* scales with *r*^2^, where r is the lens radius, while the numerical aperture scales with 1/*r*. Thus, miniature CRLs are desirable for hard x-ray microscopy at high magnifications.

Conventional microfabrication methods employed for lens patterning, including Si deep reactive-ion etching (DRIE) [12], diamond micromachining [13], and x-ray lithography, have provided lens radii down to a few micrometers [14, 15]. However, inherent limitations of conventional microfabrication techniques meant that they only make cylindrical-focusing CRLs, thus requiring two groups of perpendicular lenses to achieve spherical focusing, thereby doubling the length of the CRL. Although not an issue for focusing a collimated beam into a spot, for microscopy this limitation halves the imaging field of view (FOV), since the FOV scales inversely with the total length of the CRL. Spherically focusing CRLs of larger diameters have been made with imprinting and bubble-in-capillary fabrication techniques [5] [10], but the focal length will be beyond 10 cm for 10s of keV x-rays, thus limiting the level of magnification in a benchtop setup.

Recently, a nano-printing process based on two-photon photopolymerization has been explored to produce a CRL for imaging with 9.25 keV x-rays at a 10 cm focal length [15], which demonstrated the ability to focus a collimated beam into a bright spot. However, there is no such lens yet for hard x-rays, particularly of shorter focal lengths for high-magnification microscopy. Here, we report the application of two-photon photopolymerization to the development of a 1.8 mm long polymer CRL with a focal length of 21.5 mm at 22 keV, and numerical aperture (NA) of 2.79x10^-4^. The resulting polymer CRL was successfully used in a benchtop microscope to image and evaluate the focal spots of tungsten anode x-ray tubes at 74x magnification.

Traditionally CRLs are used with monochromatic sources, since the focal length of the same CRL varies with x-ray wavelength due to the spectral dispersion of the refractive index of the CRL material. In contrast, in this study we imaged the focal spots of tungsten-anode x-ray tubes which had finite bandwidth. Thus, for a given focal spot-to-lens distance, there was a specific wavelength that provided an in-focus image of the focal spot, combined with other wavelengths that gave out-of-focus images of the focal spot. As in the appearance of objects in a consumer camera that are either in front of or behind the focal plane, an out-of-focus image is the convolution of a sharp image with a broadened, out-of-focus point spread function of the lens. Therefore, an out-of-focus image of the focal spot will be enlarged relative to an in-focus image. With the finite bandwidth of the x-ray tubes, the overall image was a combination of both in-focus and out-of-focus images. Consequently, the apparent focal spot size from the images provided an assessment of the extent of out-of-focus broadening, and the ability of the CRLs to function with such sources. The actual system resolution with a broadband source was also measured in a standard blade edge measurement.

## Materials and methods

### Design

For a CRL made of a series of concave lenses each having a parabolic surface, the focal length is:

$$f=\frac{r^{2}}{2Nl\delta }$$ (1)

Where the refractive index decrement *δ* = 1 − *n*, *r* is the radius of the parabola, *l* the length of an individual parabolic surface, and *N* the number of lenses.

**Fig 1:**
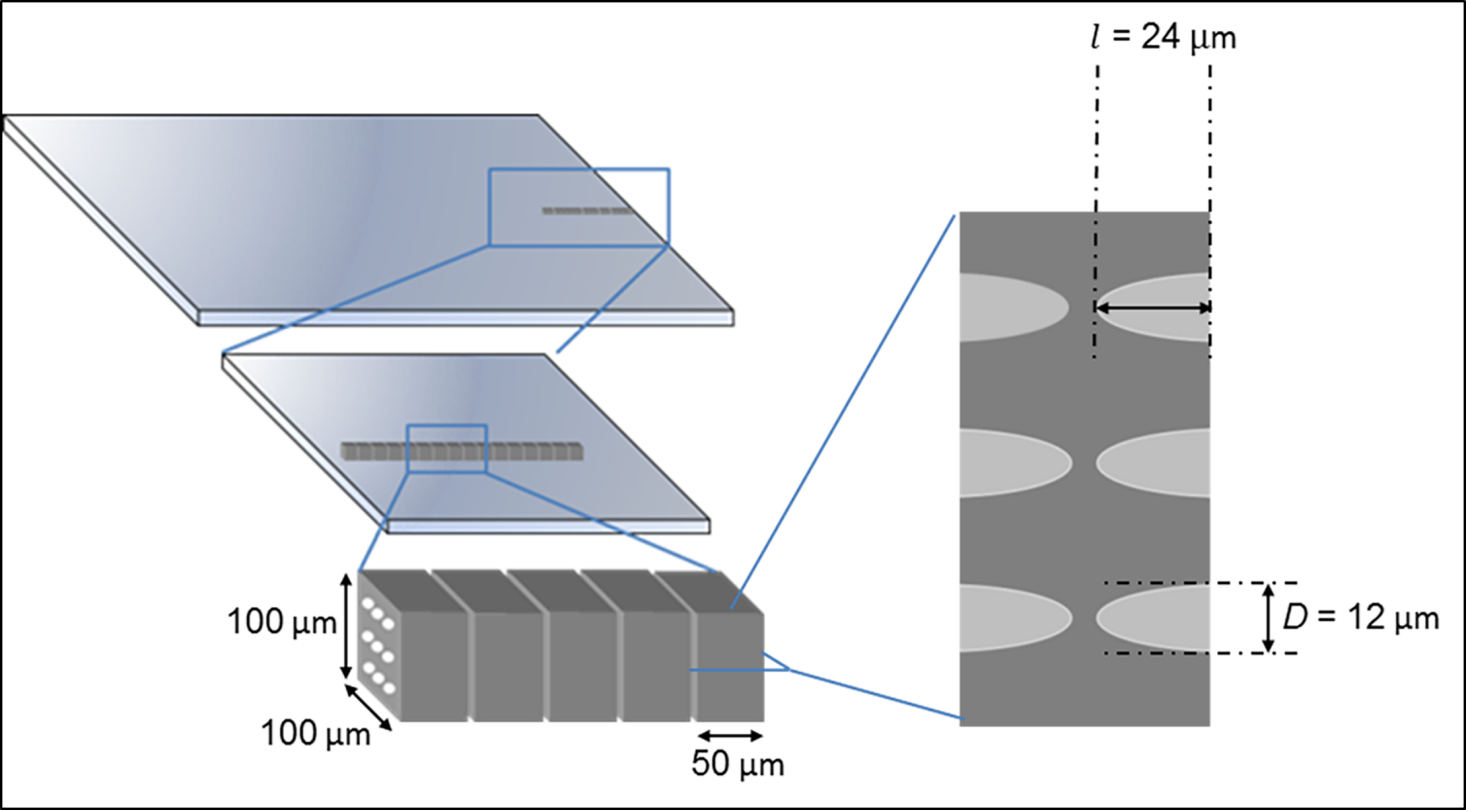
Schematic of the spherically focusing CRL. The CRL consists of 32 in-line blocks with 7 μm gaps between them. Each block is 100 μm tall by 100 μm wide by 50 μm long, and contains an array of 3x3 parabolic indents at both the front and back. Each parabolic surface indent has a depth l of 24 μm and a radius r of 6 μm. The design provides 3x3 replicate CRL columns, giving 3x3 replicate images in the microscopy set up.

Our parabolic CRL consists of 32 in-line blocks with 7 μm gaps (Fig 1). Each block is 100 μm tall by 100 μm wide by 50 μm long, and contains an array of 3x3 parabolic indents at both the front and back. Each parabolic surface indent has a depth *l* of 24 μm and a radius *r* of 6 μm. This design provided 3x3 replicate CRL columns, giving 3x3 replicate images in the microscopy set up to allow measurements of variability among them. Based on the refractive index of the photosensitive polymer material, the focal length at 22 keV according to Eq. (1) is 21.5 mm.

### Fabrication

The CRL was fabricated with the Nanoscribe 3D printer (Nanoscribe GmbH). The process is based on two-photon photopolymerization of a proprietary photosensitive polymer. The general principle of the process is that a molecule absorbs two photons simultaneously, where the first photon boosts the molecule into a virtual state for a short duration to allow it to absorb a second photon to reach the final state, with an energy increment that is twice that of a single photon. This process is driven by a focused femtosecond laser. Specifically, polymerization occurs when the weakly cross-linked polymers break by the absorption of the photons and generate two radicals, which will react with monomers. Radicalized monomers will react with each other and create three dimensional highly cross-linked polymers. The polymerization is localized at the laser focal point. The focal point is scanned in 3D space to write the designed structure. The unpolymerized part is then removed by solvent after laser exposure. The process enables high-resolution patterning below the diffraction limit of the laser beam [16].

**Fig 2:**
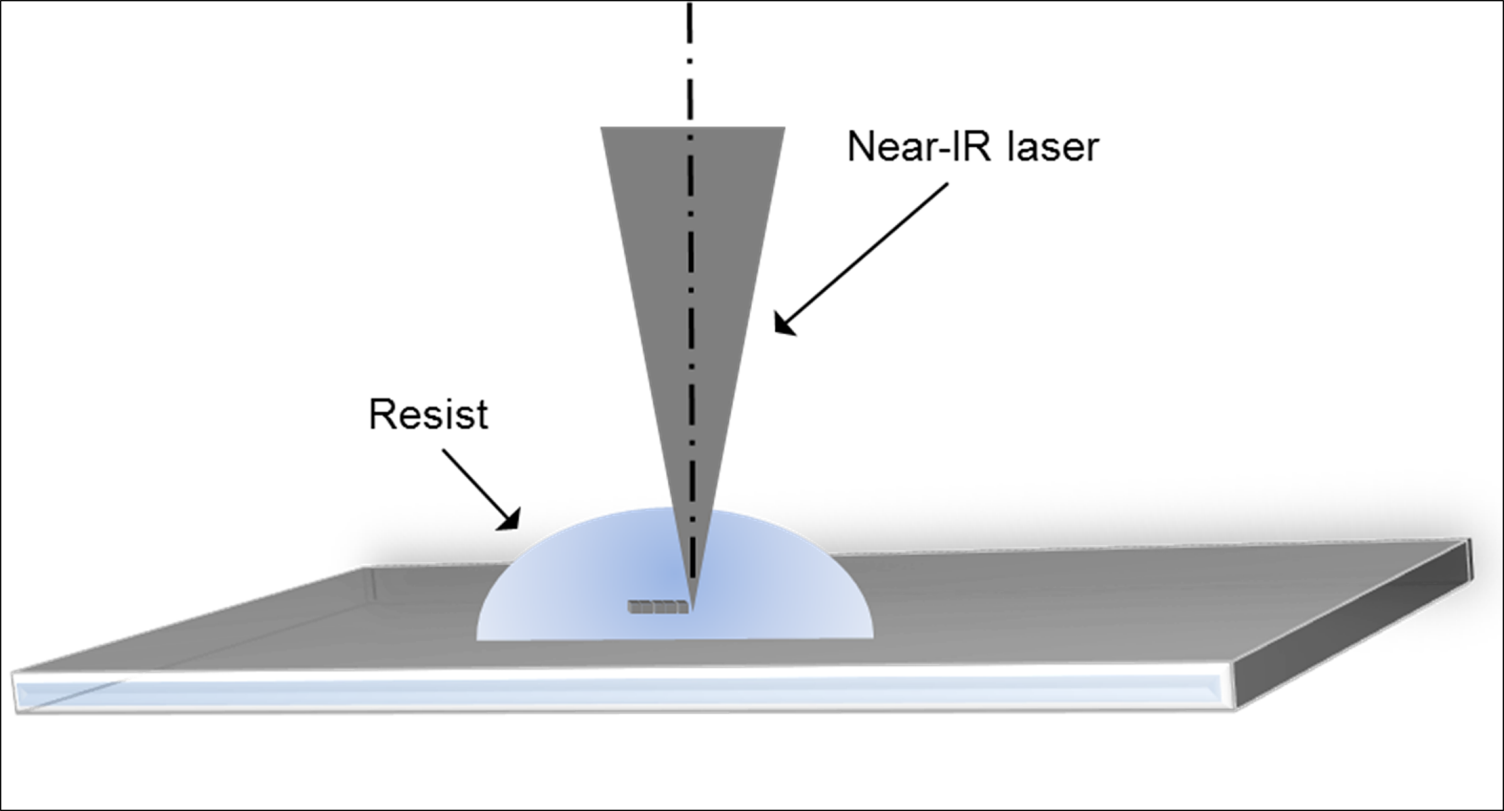
Illustration of the dip-in laser lithography (DILL) mode. The polymer CRL was printed in the DILL mode of laser scanning.

The specific configuration used to print the CRL is the dip-in laser lithography (DILL) mode of the Nanoscribe system (Fig 2). In this mode, a droplet of IP-DIP resist was drop-casted on the center of a 700 μm thick, 25 mm square fused silica substrate. The CRL elements were patterned in the resist via layer-by-layer scanning of the laser focal spot at 400 nm resolution. The writing process for a typical device was completed in approximately 2 hr. The substrate was then immersed in a propylene glycol methyl ether acetate developer for 15 min, followed by a 15 min bake at 70°C. A shadowing effect of the laser beam that occurred when writing separate blocks next to each other was eliminated by maintaining a minimum gap of 7 μm between adjacent blocks in the array. Other processing conditions such as laser power, layer thickness and hatching distance were also crucial in making a robust structure without distortion. After characterizing the process with a number of trials, we found that a suitable lens structure could be achieved using a hatching distance and layer thickness of 400 nm and a laser power level of 30 mW, which was 60% of the maximum power of 50 mW.

### Imaging x-ray tube focal spots and measurement of imaging system resolution

**Fig 3:**
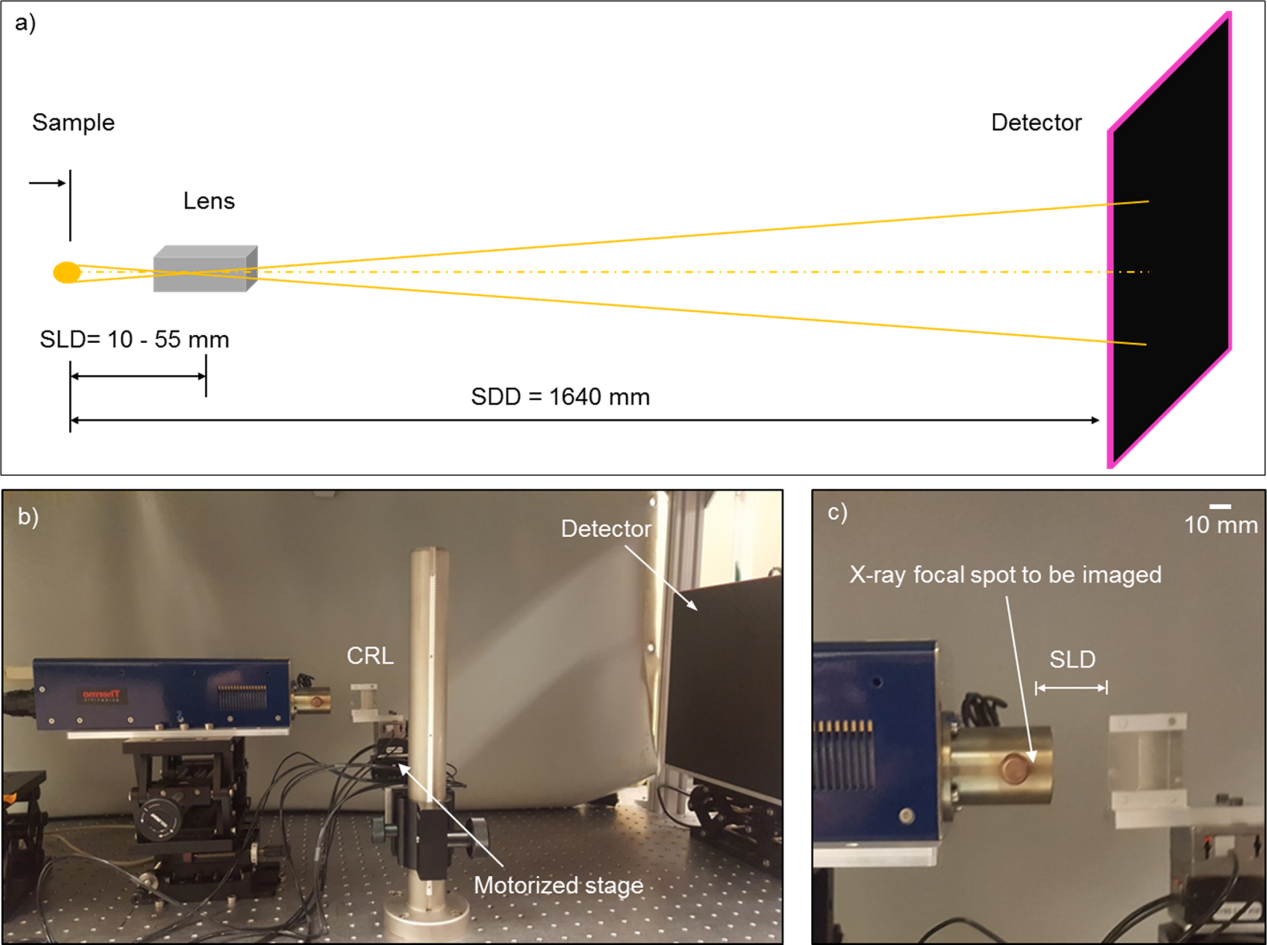
Imaging the focal spots of X-ray sources. **a)** A schematic of the x-ray microscopy setup for imaging the focal spots of x-ray sources. The focal spots are light emitting samples and do not need additional illumination. The CRL is mounted on a motorized stage with all 6 degrees of movement and rotation, allowing the sample-to-lens distance (SLD) to be varied between 9.8 mm and 54.8 mm, and alignment of the lens axis with the beam. The sample-to-detector distance (SDD) is fixed. Geometric magnification is given by the ratio (SDD-SLD)/SLD, which ranged between 166 and 28.9. **b)** In this photograph of an imaging experiment, the focal spot of a tungsten-target micro-focus source was being imaged. The silica substrate of the CRL was mounted vertically on the motorized stage. The detector was moved closer to the CRL from its working position to fit into the photograph. **c)** A closer view of the x-ray tube window and the silica substrate of the CRL illustrate the SLD.

A fabricated polymer CRL was integrated into a benchtop setup to image the focal spots of x-ray sources (Fig 3). The setup consisted of an in-line arrangement of the x-ray source, the CRL, and a flat panel detector (Fig 3A). Since the imaged sample was the x-ray focal spot itself, no additional illumination was needed. The CRL was positioned at a variable sample-to-lens distance (SLD) from the CRL, and a flat panel detector was fixed at a sample-to-detector distance (SDD) of 1640 mm from the x-ray focal spot. The CRL was mounted on a motorized stage with piezo micro-positioners which provided digitally-controlled movement and rotation in all 6 degrees of freedom. The motorized stage facilitated the alignment of the CRL with the beam axis. The detector (PaxScan 3024M, Varian, CA, USA) had a pixel size of 83 μm.

Two types of micro-focus x-ray sources were evaluated to determine the geometric magnification factor, given by *M* = (SDD-SLD)/SLD. The first source was a fixed-anode, tungsten-target micro focus tube (Thermo Scientific™ Kevex PXS5-928) with a vendor-specified nominal focal spot size of 4 μm at 2W power (Fig 3B and 3C). With this source, we tested the influence of two parameters on the measured focal spot size: the SLD was varied from 9.8 mm to 54.8 mm at 3 mm increments by moving the CRL along the beam, and the x-ray tube voltage was varied between 25 kV and 85 kV. The range of SLD gave a range of magnification from *M* = 166 to 28.9. The second source was a fixed-anode, tungsten-target micro focus tube (Oxford UltraBright 96000 Series) with a vendor-specified minimum focal spot size of 13 μm. In this source, we tested the stability of the focal spot size and focal spot position at 45 kV tube voltage and 6 W power setting over a period of 15 minutes. The SLD was fixed at 21.8 mm, which was the focal distance for 22 keV photons that satisfied the relationship 1/SLD + 1/(SDD-SLD) = 1/*f*. The corresponding magnification factor *M* = 74.2. Both x-ray sources had 0.254 mm Be windows, and an additional 0.25 mm Al sheet was placed on the detector to eliminate low energy photons.

Because the polymer structure had a 3x3 array of parallel CRL columns, it could provide up to 9 duplicate images of the focal spot in each shot. One or two rows of the duplicate images were obscured by the shadow of the large silica substrate when the SLD was small, resulting in between 3 to 9 usable duplicates in each shot. The average and standard deviation among the duplicate images were measured. To evaluate the focal spot size, the intensity distribution of a spot image was integrated in the y and x directions to provide x and y profiles of the spot. These were fitted to Gaussian functions to yield the full-width-half-max *FWHMx* and *FWHMy* sizes and the focal spot position (x, y). The rotationally invariant average focal spot size was calculated as FWHM = (FWHMx^2^+FWHMy^2^)^1/2^.

The overall resolution of the imaging system using a broadband tungsten-anode x-ray source was measured by imaging the edge profile of a 50 μm diameter tungsten wire. The wire was illuminated by the Oxford Ultrabright x-ray tube. In order to have sufficient magnification/pixel resolution to resolve the edge profile, the wire-to-lens distance was reduced to 10 mm to provide a magnification factor of 162 (0.51 μm de-magnified image pixel size). The x-ray tube voltage was decreased to 30 kV for shorter focal lengths. The tube power was set at 30 W, and image exposure time was 6 seconds. The position of the wire was adjusted until the wire edge appeared in the center of the image of the tube focal spot. The derivative of the edge intensity profile was fitted to a Gaussian function. The FWHM of the Gaussian fit was taken as the measured resolution of the imaging system. Twenty images were taken to provide statistics.

## Results

### Microscopy inspection of the nano-printed polymer CRL

**Fig 4:**
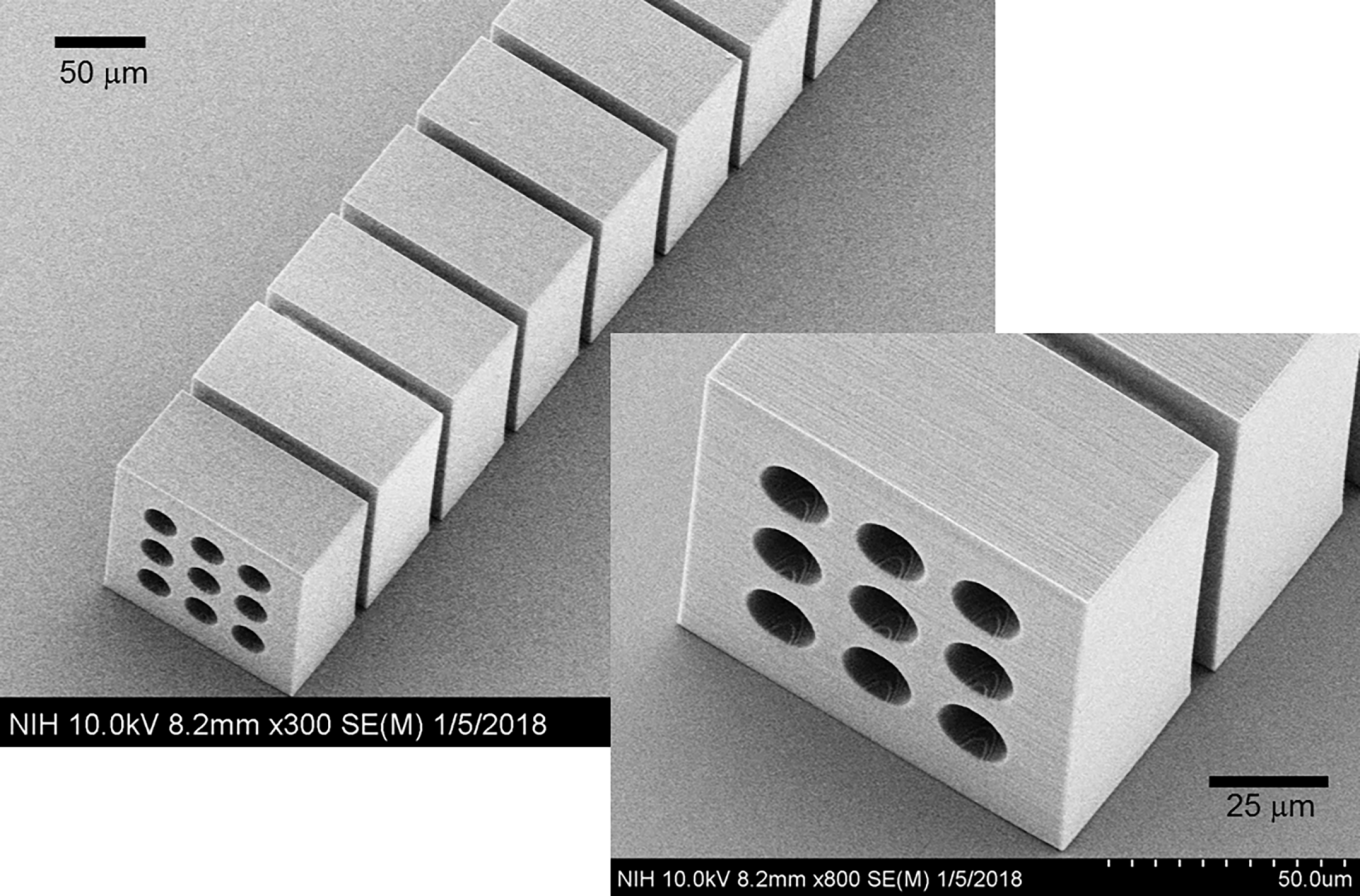
Electron micrograph of the Nano-printed CRL. The 3x3 array of concave holes are visible at one end of the CRL.

The CRL structure was inspected with scanning electron microscopy (SEM) (Fig 4). The size of individual blocks and the gap between adjacent blocks were verified. The SEM images were used to identify the Nanoscribe parameter setting that minimized the edge curvature and eliminated any visible defects on the outer surface of the blocks.

**Fig 5:**
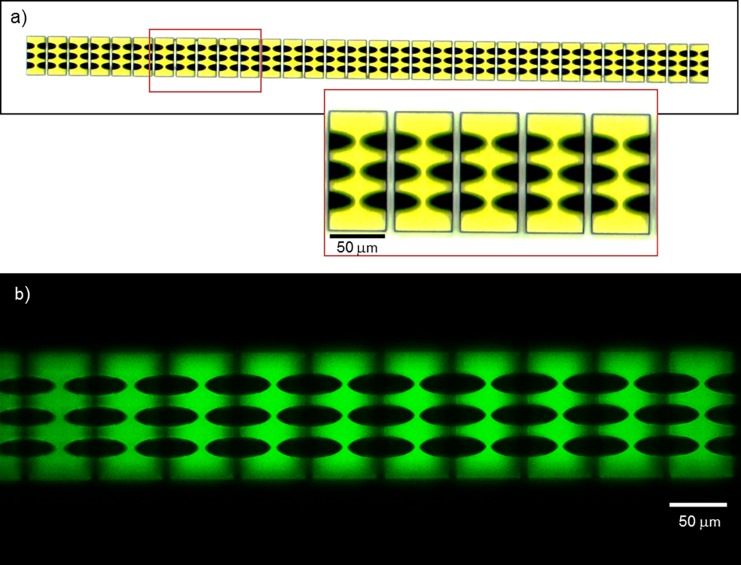
Microscopy Inspection of the Nano-printed polymer CRL. **a)** Transmitted-light bright-field optical microscopy images of the polymer CRL blocks visualizse the concave parabolic surfaces the blocks. **b)** Tw-photon fluorescence cross-sectional image at the mid-level of the first layer of parabolic concave surfaces in the blocks. Two-photon excitation was generated with an ultrafast laser at 800 nm and the fluorescence emission was recorded in the 414–535 nm range. The parabolic concave surfaces are visible against the fluorescence signal of the polymer material.

Since SEM analysis could not image below the surface of the CRL, the internal structure was inspected with light microscopy that took advantage of the optical transparency of the polymer material. Two types of optical microscopy were employed in the inspection. Fig 5A shows transmitted-light bright-field optical microscopy images of the polymer blocks (Accu-Scope 3000-LED series Microscope), where the parabolic concave surfaces were visible. The second technique that was utilized to inspect the lens internal structure was 2-photon fluorescence imaging using a 2-photon microscope (SP8, Leica microsystems) with a water immersion 1.1 NA 25x apochromat objective (CFI75 APO 25x W MP, Nikon) (Fig 5B). The sample was immersed in water, two-photon excitation was generated with an ultrafast laser (Mai Tai, Spectra Physics) at 800 nm and the fluorescence emission was recorded in the 414–535 nm range with a non-descanned hybrid detector. The excitation light was blocked before detection with a 680 nm short-pass filter (Semrock). This technique allowed depth-resolved z-sectioning of the structure and cross-sectional images of the polymer blocks. The parabolic concave surfaces of the blocks are visible against the fluorescence signal of the polymer material.

### Imaging x-ray tube focal spots and measurement of system resolution

#### X-ray focal spot analysis of the first x-ray source

**Fig 6:**
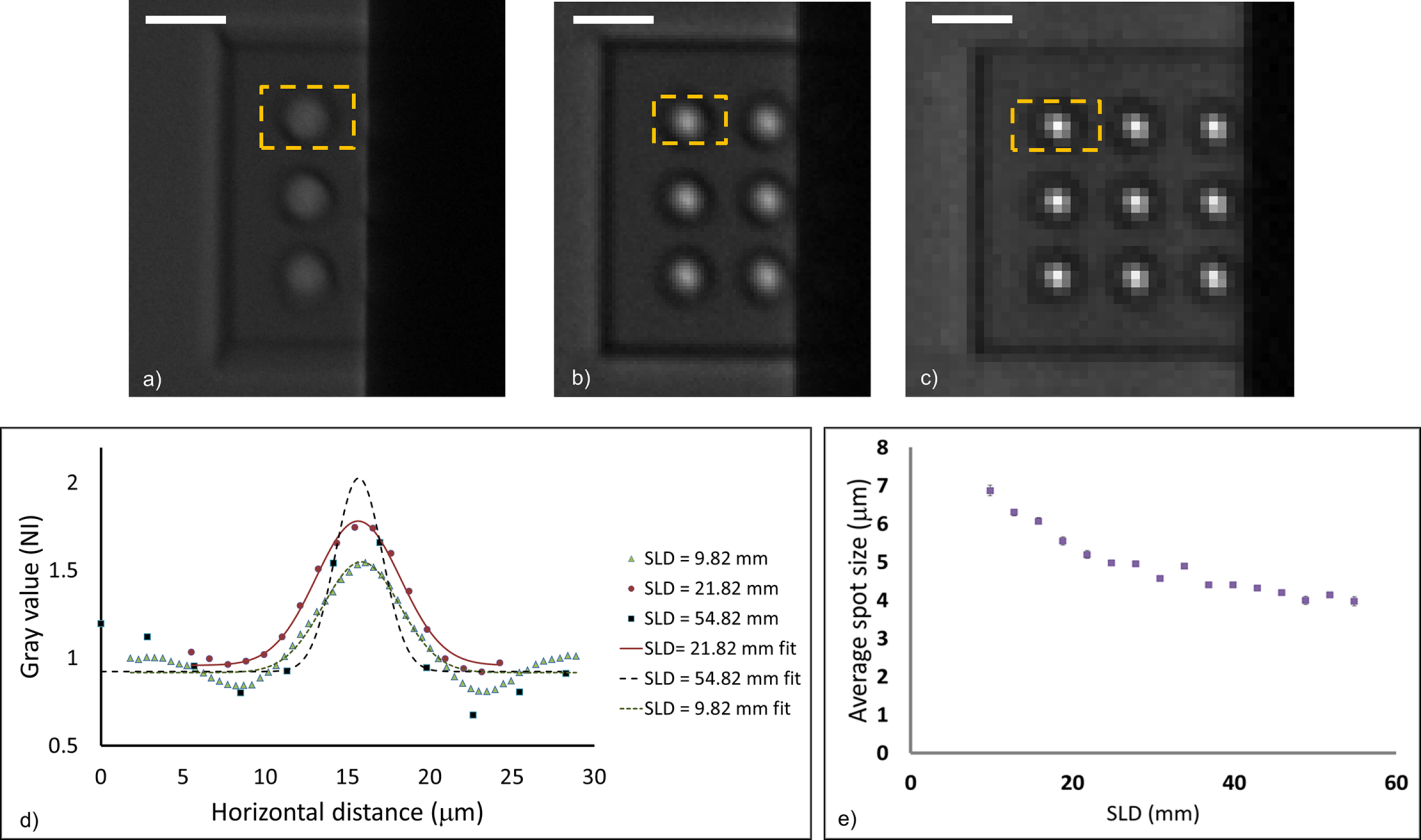
Results from the first X-ray source (Thermo Scientific Kevex) with respect to the sample-lens-distance at the source setting of 45 kV/2 W. **a)–c)** Images taken at SLDs of 9.8 mm (magnification factor M = 166), 21.8 mm (M = 74), and 54.8 mm (M = 28.9), all displayed to the same scale represented by the 25 μm scalebars. As the SLD decreased, the silica substrate on the right shadowed more of the CRLs. The pixelated appearance at low magnification (c) is due to the larger area imaged by each detector pixel. **d)** The measured intensity profiles of the spot images outlined by the yellow dotted lines in Fig 6A–6C are plotted, together with their respective Gaussian fits. Values are normalized to the baseline intensities of the images. **e)** A plot of the average measured focal spot size and standard deviation over the scanned range of SLDs. The standard deviations were < 0.14 μm for all measurements.

The first micro-focus x-ray source (Thermo Scientific Kevex) was studied at a range of tube kV settings and a range of sample-to-lens distances (SLDs) in the imaging setup. Results with respect to the SLD parameter are summarized in Fig 6 for the tube setting of 45 kV/2 W power. Images taken at SLDs of 9.8 mm (magnification factor *M* = 166), 21.8 mm (*M* = 74), and 54.8 mm (*M* = 28.9) are shown in Fig 6A–6C. It was found that as the SLD decreased, the silica substrate shadowed more of the CRLs. At the other end with large SLDs, the images appeared pixelated due to the lower magnifications and larger areas imaged by each detector pixel (Fig 6C). Since the polymer material between the CRLs was essentially transparent to x-rays, there was background intensity between the focal spot images, with each spot surround by a dark halo that represented the projected area of an individual CRL column. The measured horizontal profiles of the spot images outlined in Fig 6A–6C are plotted in Fig 6D, together with their respective Gaussian fits. Fig 6E is a plot of the average measured focal spot size and standard deviation over the scanned range of SLDs. It was found that at 45 kV and 2 W power, the measured spot size decreased from 6.8 ±0.14 μm at the smallest SLD to 4.0 ±0.12 μm at the large SLD. At the SLD of 21.8 mm, which was the focal distance for 22 keV photons, the measured spot size was 5.17 ±0.01 μm. The trend of measured spot size with SLD is discussed in the Discussion section below.

**Fig 7:**
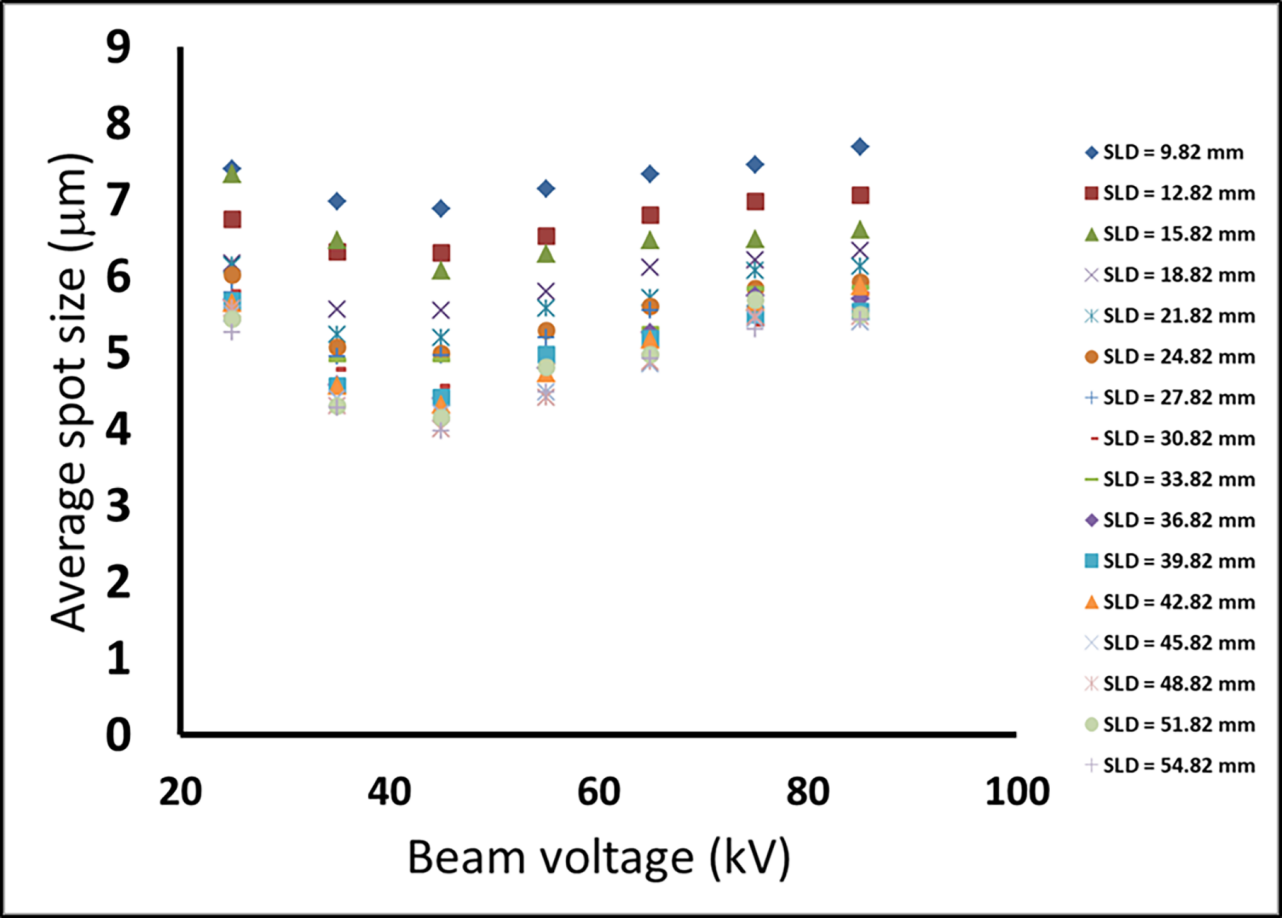
Measured average focal spot size of the first X-Ray source (Thermo Scientific Kevex) as a function of the X-Ray tube voltage for All SLDs. The measured focal spot size was consistently the smallest at 45 kV tube voltage for all SLDs. The standard deviations of the measurements were all less than 0.22 μm.

Results from the first micro-focus x-ray source with respect to the tube kV setting are summarized in Fig 7. At each SLD, the average measured focal spot size over from 25 kV to 85 kV at the 10 kV increment was graphed. It was found that the measured focal spot size was consistently the smallest at 45 kV tube voltage for all SLDs. The interpretation of this minimum is addressed in Discussion.

#### X-ray focal spot analysis of the second x-ray source

**Fig 8:**
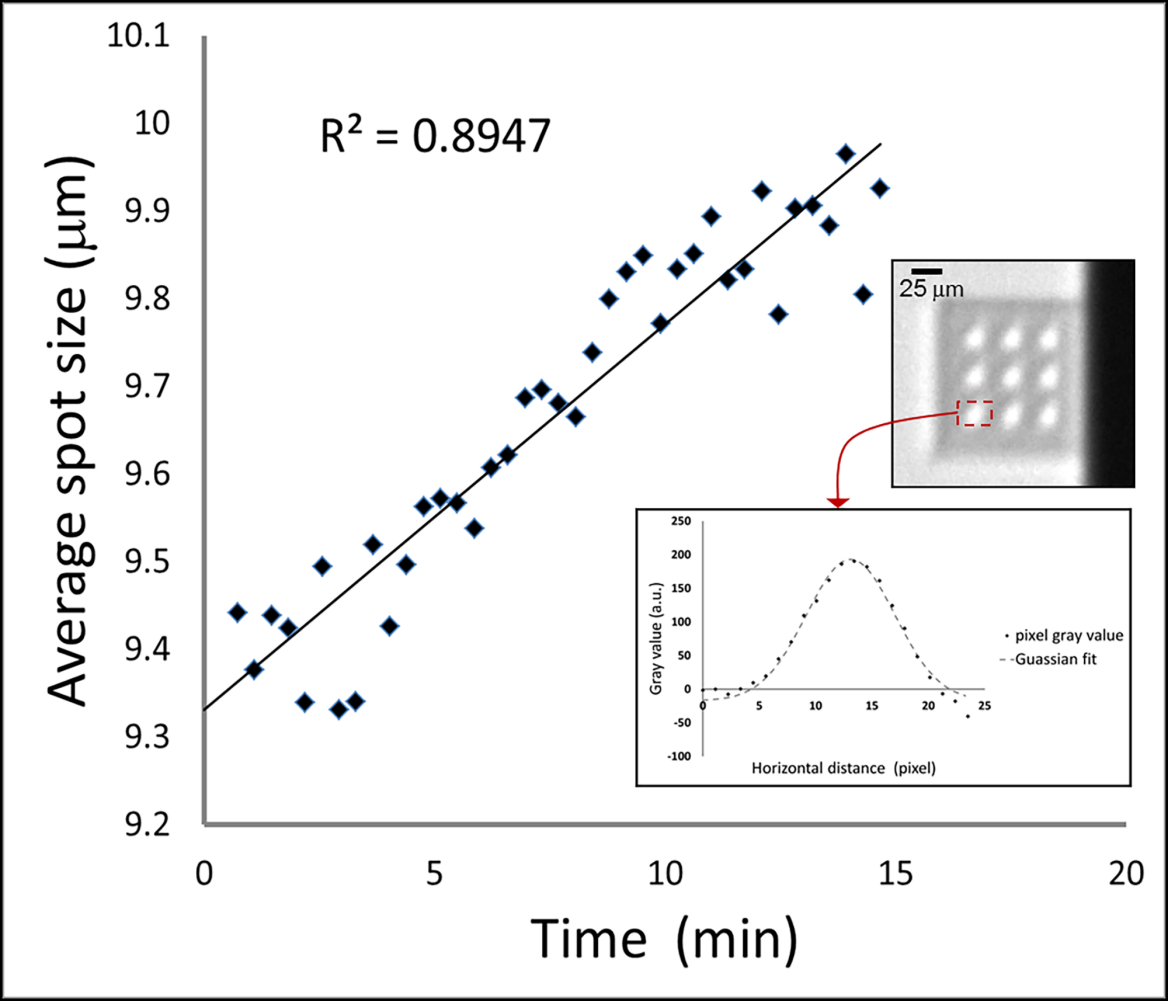
Average focal spot size over time for the second X-Ray source operating at 45kV/6W. The upper inset is an example shot of a 6 second exposure which contained 9 duplicate images of the focal spot from the 3x3 array of CRL columns. The lower inset is the x intensity profile of a spot and its Gaussian fit. The main plot is the average FWHM of the 9 duplicate spot images as a function of time. The average spot size fluctuated by 0.1 μm at each time point and expanded by 5.5% over the 15 minute duration.

Imaging of the second micro-focus x-ray source focused on the stability of the size and position of the focal spot when operating at 45 kV and 6 W power. Results of the focal spot size measurements are summarized in Fig 8. Fig 8 includes an example shot containing 9 duplicate images of the focal spot from the 3x3 array of CRL columns. It also shows the line profile of the spot intensity in the x direction, and the Gaussian fit of the profile to quantify its FWHM and peak position. The average spot size of the 9 duplicate images as a function of time was plotted with a linear regression line of R^2^ = 0.89. The measured spot size fluctuated by 0.1 μm at each time point and expanded gradually by 5.5% over the 15 minute period, from 9.44 μm to 9.96 μm.

**Fig 9:**
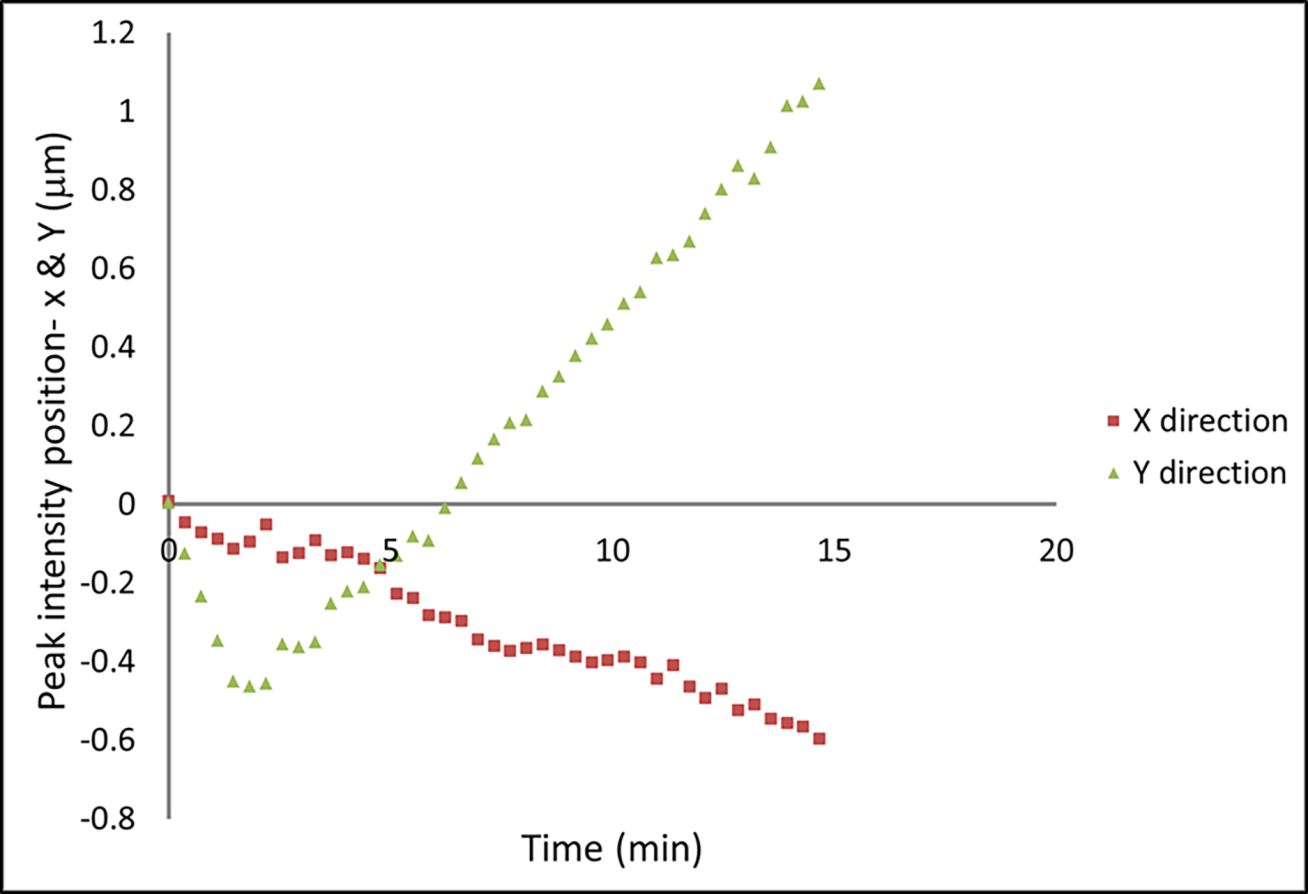
Drift of the focal spot position of the second X-ray source over a period of 15 minutes in X and Y directions. Each measurement is the average of the 9 duplicate images of the focal spot from the 3x3 array of CRL columns. The standard deviations of the measurements were < 0.1 μm.

Data revealing drift of the focal spot position are graphed in Fig 9. Since the image taken at each time point contained 9 duplicate images of the focal spot, the peak positions of the 9 spots were individually determined with Gaussian fits, and relative displacements of the peak positions from the first shot were averaged over the 9 duplicates. The average x and y displacements over time are plotted in Fig 9. It illustrates that the focal spot moved unidirectionally in the horizontal direction by 0.6 μm. In the vertical direction, however, the focal spot initially moved down for 0.5 μm in a 2 minute period, then reversed direction and moved up 1.6 μm over the remaining 13 minutes.

#### Measurement of imaging system resolution

**Fig 10:**
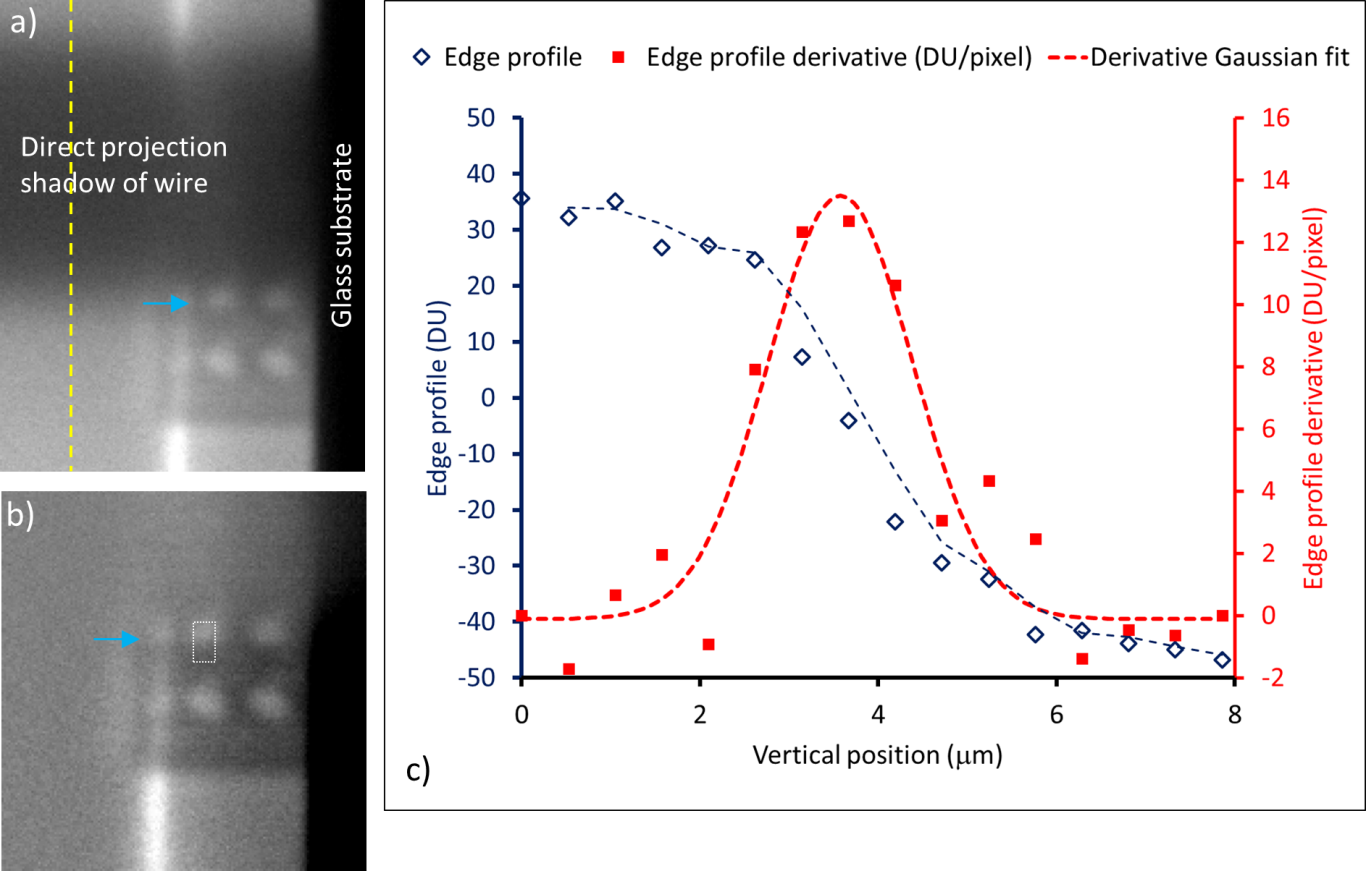
Measurement of the imaging system resolution by the edge profile method. **a)** In the raw image, the highly magnified and blurred shadow is the direct projection of the horizontal tungsten wire. The lower edge of the wire was positioned to cross the central row of CRLs. Within the two central CRLs, sharpened and inverted images of the wire edge is visible due to focusing by the lenses (vertical position indicated by the blue arrow). The rightmost column of CRLs were obscured by the shadow of the glass substrate. The background intensity profile outside the CRL block down the green dotted line was subtracted from every pixel column of the image before data analysis. **b)** After subtracting the background direct projection profile of the wire, only the inverted images of the wire edge within the two CRLs are visible. The vertical position of the edge is indicated by the blue arrow. The intensity profile of the edge highlighted by the rectangle is plotted in c). **c)** The edge profile, derivative of the profile and Gaussian fit of the derivative are plotted. The FWHM of the Gaussian fit measures the resolution of the imaging system as a whole. From 20 such images the system resolution was determined to be 2.30±0.22 μm.

Fig 10 is a collection of the images, the intensity profile of the magnified tungsten wire edge through a CRL, the derivative of the edge profile and its Gaussian fit. In the raw image of Fig 10A, direct projection of the horizontal tungsten wire gave a highly magnified and blurred shadow which is superimposed on the images of the tube focal spot within the CRL block. The lower edge of the wire was positioned to cross the central row of CRLs. Within the two central CRLs, sharpened and inverted images of the wire edge is visible due to focusing by the lenses (vertical position indicated by the blue arrow). The rightmost column of CRLs were nearest to the glass substrate and obscured by the shadow of the substrate. The bright vertical stripe down the middle of the image is reflected x-rays from the surface of the glass substrate. In Fig 10B, the direction projection profile of the wire was subtracted, leaving only the inverted images of the wire edge within the two CRLs. The vertical position of the edge is indicated by the blue arrow. The intensity profile of the edge highlighted by the rectangle is plotted in Fig 10C. The derivative of the profile is also plotted together with a Gaussian fit. The FWHM of the Gaussian fit measures the resolution of the imaging system as a whole. From 20 such images, the measured system resolution was 2.30±0.22 μm (average and standard deviation).

## Discussion

This work demonstrated that sub-micron 3D printing based on two-photon photopolymerization is suitable for realizing compound refractive lenses for hard x-rays. The fabricated CRL devices offer the smallest size and shortest focal length that has been reported in the literature for operation with photons in the range of 10s of keVs.

The resolution of the CRL imaging system using broadband x-ray tubes is determined by the widened point-spread-functions of the parts of the spectrum that are out of focus for a particular sample-to-lens distance. This is why the measured resolution of 2.3 μm at 30 kV tube voltage was substantially larger than the 0.1 μm Fresnel diffraction width associated with a monochromatic photon energy of 15 keV. The width of the x-ray spectrum is dependent on the tube voltage setting, and so is the out-of-focus broadening of the resolution. Therefore, over the tube voltage range of 25 kV to 85 kV, the system resolution may vary, and contributes to the energy dependence of the size of the focal spot image as plotted in Fig 7. If the polymer CRL is coupled to a quasi-monochromatic source, for example the characteristic line emissions of metal-anode x-ray tubes used in prior CRL studies [2][15], it can reasonably be expected to provide sub-micrometer resolutions.

The industry standard for direct imaging of x-ray tube focal spots is by pinhole cameras, where the size of the pinhole combined with its Fresnel diffraction width is the image resolution. Therefore, to obtain the same resolution as reported here, the pinhole would have a diameter of 2.3 μm, resulting in a flux through the pinhole equal to 1/27^th^ the flux through the diameter of the CRL. Correspondingly, the exposure time to obtain the same level of image intensity would be 27 times the 6 second exposure in our experiments, or 2.7 minutes long. Therefore, when compared to a pinhole camera, the advantage of the CRL is the ability to measure focal spot drifts on the time scale of seconds, which would otherwise appear as an artefactual spot elongation in an exposure of minutes. However, the 3D printed polymer CRLs lack the light-blocking aperture of traditional lenses which leads to a substantial background intensity, negating the advantage of the higher flux throughput. Therefore, a future direction of development is the removal of the background intensity by adding apertures at both ends of the CRL which match the diameter of the micro lenses. The main engineering challenge is expected to be the alignment and fixture of the apertures with the CRL.

**Fig 11:**
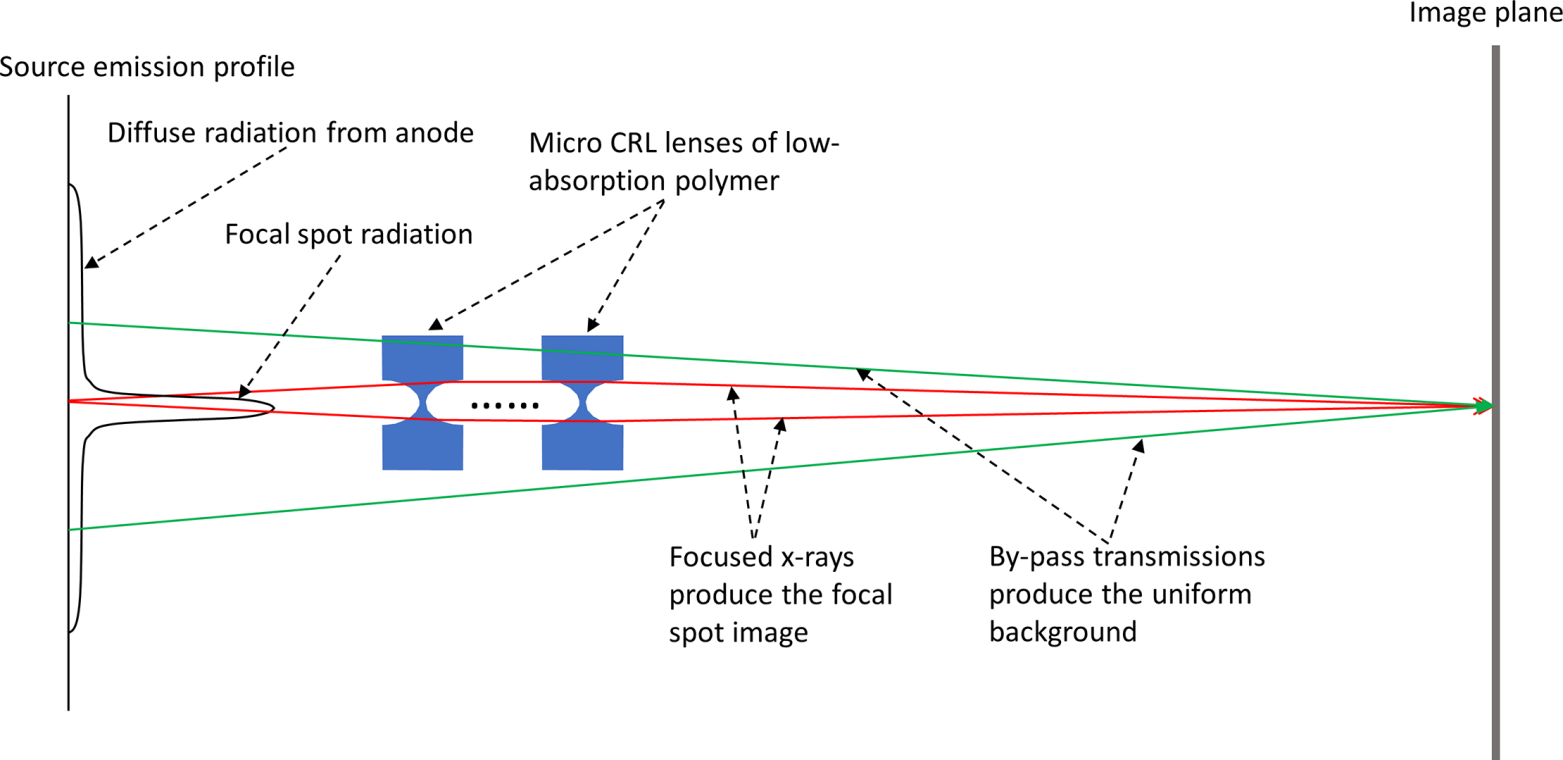
Illustration of the source of the background intensity in the polymer CRL-based images. Due to the transparency of the polymer to hard x-rays, a location on the image plane receives focused x-rays which produce the image of the x-ray tube focal spot, and x-rays from the broad diffuse radiation around the focal spot that by-pass the lenses which produce the uniform intensity background seen in Fig 6.

Referring to Fig 6A–6C, there was a uniform background intensity throughout the polymer area both between and outside the CRLs, and in all areas outside the polymer blocks that were not covered by the thick glass substrate. Unlike the out-of-focus effect from the broad spectrum of the x-ray tubes, the background comes from light transmission that by-passes the lenses (Fig 11). With a conventional lens surrounded by a light blocking aperture, for example, a microscope objective in a metal housing, there is no such by-pass transmission. Consequently, there is no uniform background intensity regardless of whether the lens is in focus or out of focus, owing to the fact that the point-spread-function of a lens always has a finite size and drops off rapidly to zero beyond that size [17]. In fact, the point-spread-function of a conventional lens of the same size as our CRLs would be a 12 μm disk in the worst-case scenario of no focusing, which would not have produced the uniform background seen in these images. However, the polymer CRLs are surrounded by the polymer material itself, which allows x-rays to pass with about 15% attenuation at the thickest part. Consequently, the same location on the detector receives focused light that forms the image, and by-pass transmission from diffuse radiation of the source that forms the elevated background (Fig 11). This notion is supported by the observation of the same background level regardless of the changing focusing conditions with the sample-to-lens distance (Fig 6). In prior imaging study with macro CRLs, the aperture condition was met by light-absorbing walls around the diameter of the CRL [2].

Several questions emerge from the presented experimental data. The measured focal spot size of the first x-ray source at 45 kV decreased gradually with increasing SLD, even beyond the designed focal distance (Fig 6). We speculate two possible reasons for the trend. One is that at large SLDs/low magnification, each detector pixel covers a larger area of the focal spot, leading to increasing under-sampling of the spot profile (Fig 6C) and possible systematic under-estimation of the width of the underlying profile. Another possible reason was that the higher parts of the x-ray spectrum (> 22keV) continually come into focus with increasing SLD.

In the study of focal spot size vs. tube kV setting, the measured spot was the smallest at 45 kV at all SLD values. There are two possible explanations for this observation. One is that the x-rays were over-focused at < 45 kV and under-focused at > 45 kV, while another is that the x-ray focal spot size was actually voltage dependent. Considering the fact that the minimum was consistent for all SLDs, the data dispute the first explanation, and are more consistent with an actual voltage dependence of the focal spot size.

The field of view (FOV) of the polymer CRL is determined by the acceptance angle of the string of lenses in the CRL, rather than the diameter of each lens: the acceptance angle is the angle spanned by the radius of the front lens relative to the iso-center of the CRL [2]. From any point within this acceptance angle, the majority of light that enters the front lens of the CRL will also pass through the entire string of lenses and experience the full focusing effect. The acceptance angle is expressed as 2*r*/*L*. For a given sample-to-lens distance, the FOV is the acceptance angle multiplied by that distance. the sample-to-lens distance for high magnification setups is close to the focal length f, therefore, the FOV is expressed as 2r*f/*L*. For these micrometer-scale CRLs, the ratio of *f*/*L* is greater than 10, and therefore, the FOV is estimated to be 145 μm and much greater than the lens diameter itself. The geometry for these CRLs resembles more the situation of a pin-hole camera than the situation of a microscope objective lens. It is worth noting that the FOV spans only a small angle of ±0.19° off the lens axis, which is not expected to incur significant off-axis aberration with parabolic lenses [4]. The FOV was tested experimentally by observing the images of the x-ray focal spot at various off-axis distances. It was observed that the amplitude and FWHM of the focal spot images were preserved over a ±75 μm lateral offset.
